# Induction of vasculogenesis in breast cancer models

**DOI:** 10.1038/sj.bjc.6600610

**Published:** 2002-11-26

**Authors:** K Shirakawa, S Furuhata, I Watanabe, H Hayase, A Shimizu, Y Ikarashi, T Yoshida, M Terada, D Hashimoto, H Wakasugi

**Affiliations:** Pharmacology Division, National Cancer Center Research Institute, Tsukiji 5-1-1, Chuo-Ku, Tokyo 104-0045, Japan; Genetics Division, National Cancer Center Research Institute, Tsukiji 5-1-1, Chuo-Ku, Tokyo 104-0045, Japan; Department of Surgery, SaitamaMedical Center, SaitamaMedical School, 981 Tsujido-machi, Kamoda, Kawagoe-shi, Saitama 350-8550, Japan

**Keywords:** endothelial cells (ECs), endothelial precursor cells (EPCs), breast cancer, angiogenesis, vasculogenesis

## Abstract

Recently, there have been reports of postnatal vasculogenesis in cases of ischaemia models. The aim of the present study is to provide evidence of postnatal vasculogenesis in breast-cancer–bearing mice. Based on cell surface antigen expression, we isolated endothelial precursor cells from bone marrow, peripheral blood and tumour-infiltrating cells from mice that had received six human breast cancer xenografts. In all three areas (bone marrow, peripheral blood and tumour-infiltrating cells), endothelial precursor cell population was elevated in all transplanted mice. Differentiation and migration activities of endothelial precursor cells were measured by comparing levels of the endothelial precursor cell maturation markers Flk-1, Flt-1, Tie2, VE-cadherin and CD31 among these three areas. The endothelial precursor cell population was 14% or greater in the gated lymphocyte-size fraction of the inflammatory breast cancer xenograft named WIBC-9, which exhibits a hypervascular structure and *de novo* formation of vascular channels, namely vasculogenic mimicry (Shirakawa *et al*, 2001). *In vitro*, bone marrow-derived endothelial precursor cells from four human breast cancer xenografts proliferated and formed multiple clusters of spindle-shaped attaching cells on a vitronectin-coated dish. The attaching cells, which incorporated DiI-labelled acetylated low-density lipoprotein (DiI-acLDL) and were negative for Mac-1. The putative bone marrow derived endothelial precursor cell subset, which was double positive of CD34 and Flk-1, and comparative bone marrow derived CD34 positive with Flk-1 negative subset were cultured. The former subset incorporated DiI-acLDL and were integrated with HUVECs. Furthermore, they demonstrated significantly higher levels of murine vascular endothelial growth factor and interleukin-8 in culture supernatant on time course by enzyme-linked immunosorbent assay. These findings constitute direct evidence that breast cancer induces postnatal vasculogenesis *in vivo*.

*British Journal of Cancer* (2002) **87**, 1454–1461. doi:10.1038/sj.bjc.6600610 www.bjcancer.com

© 2002 Cancer Research UK

## 

Postnatal neovascularisation is thought to result exclusively from proliferation and remodelling of fully differentiated ECs derived from pre-existing blood vessels (Holash *et al*, 1999). This adult paradigm, referred to as angiogenesis, contrasts with vasculogenesis, the term applied to the formation of embryonic blood vessels from endothelial precursor cells (EPCs). Recently, however, EPCs have been isolated from adult peripheral blood (PB) and umbilical cord blood, and these cells attach themselves to vitronectin-coated dishes (Asahara *et al*, 1997; 1999; Isner and Asahara, 1999; Takahashi *et al*, 1999; Kalka *et al*, 2000; Murohara *et al*, 2000). Augmentation of transfused EPCs has been observed in ischaemic tissue, and participation of transfused EPCs in neovascular formation has been observed in mature animal models. Previously, we reported the establishment of a new human IBC xenograft (WIBC-9) that exhibits angiogenesis and *de novo* formation of vascular channels, vasculogenic mimicry (Shirakawa *et al*, 2001). WIBC-9 overexpresses human (h) angiogenic factors (interleukin-8 (IL-8), vascular endothelial growth factor (VEGF), basic fibroblast growth factor (bFGF), angiopoietin-1 (Ang-1)) and murine (m) angiogenic factors (integrin αvβ3, flt-1, tie-2, VEGF, Ang 2), compared with 3 non-IBC xenografts, at the mRNA and/or protein level. Previously, by blocking the VEGF-Flt-1 and Angiopoietin 1,2-Tie2 pathways in IBC, we achieved a ratio of tumour growth inhibition of 99% or greater and demonstrated marked anti-angiogenic and putative anti-vasculogenic effects (Shirakawa *et al*, 2002). In the present study, we tested our hypothesis that human breast cancer lines (specifically, WIBC-9) can induce proliferation, of ECs and EPCs in animal models.

## MATERIALS AND METHODS

### Human VEGF transfection vector (pcDNA3-hVEGF) and cell lines

An *Eco*RI-*Hind*III 0.6 kb human(h)VEGF cDNA fragment was subcloned into the pcDNA3.1/Neo vector (Invitrogen, San Diego, CA, USA). SK-BR3 and MCF-7 cells (American Type Culture Collection, Rockville, MD, USA) grown in DMEM with 10% FBS were transfected with this expression construct (pcDNA3.1/hVEGF) or with the vector alone (pcDNA3.1/Neo) using the Lipofectamine Plus reagent (Invitrogen, San Diego, CA, USA) and selected with Geneticin(G418) (Invitrogen, San Diego, CA, USA). Stably-transfected SK-BR3/hVEGF and MCF-7/hVEGF cell lines were maintained in media containing 400 μg ml^−1^ Geneticin.

### Xenografts

WIBC-9 and the established non-IBC xenografts MC-2, MC-5 and MC-18 were maintained as described elsewhere (Shirakawa *et al*, 2001). SK-BR3, MCF-7, SK-BR3/hVEGF and MCF-7/hVEGF cells were injected subcutaneously into the second mammary fat pads of athymic, female, ovariectomised 4-week-old BALBc nu/nu mice (10^7^ cells 100 μl^−1^ serum-free culture medium). To evaluate EPCs and hVEGF concentration in peripheral blood, retro-orbital bleeding into capillary tubes was undertaken from tumour-bearing mice (*n*=5 per study group) when the tumour became 10 mm in maximum diameter after transplant. Femurs were also undertaken from these mice to extract bone marrow. All animal experiments have been carried out with ethical committee approval. The ethical guidelines that were followed meet the standards required by UKCCCR guidelines (Workman *et al*, 1998). WIBC-9 and the established non-IBC xenografts MC-2, MC-5 and MC-18 were maintained as described elsewhere (Shirakawa *et al*, 2001).

The animals received a weekly percutaneous administration of 100 μg of 17β estradiol (Sigma, Saint Louis, MO, USA) in 10 μl of ethanol, in order to obtain tumours with a volume of 1000 mm^3^.

### Mobilisation of ECs and EPCs by recombinant human GM–CSF (rhGM–CSF) and rhVEGF

rhGM–CSF (molecular weight, 14 kD) and rhVEGF (molecular weight, 38.2 kD), in powdered form (Sigma), were dissolved in phosphate-buffered saline (PBS). To determine the mobilisation activity of GM–CSF and VEGF on ECs and EPCs, 50 ng of rhGM–CSF or 10 ng of rhVEGF was injected into BALB/c nude mice (Clea Japan, Tokyo, Japan) intraperitoneally as *in vivo* controls.

### Preparation of cultured conditioned medium and determination of hVEGF concentration by ELISA

SK-BR3, SK-BR3/hVEGF, MCF-7 and MCF-7/hVEGF cells (1×10^6^ cells well^−1^) were incubated for 2 days at 37°C, and the supernatants were then collected and stored at −80°C until used. Concentration of hVEGF was measured by ELISA in 100 μl samples of supernatant, using immunoassay kits (Immuno-Biological Laboratories Co., Ltd., Fujioka, Japan). Each assay was performed in triplicate.

### Flow cytometry (quantitation of EPCs and ECs)

To examine quantity and differentiation of EPCs, mononucear cells (MNCs) (derived from BM, PB and TI cells) from each xenografted BALB/c nude mouse (*n*=5) were subjected to flow cytometric analysis to examine surface expression of the proteins mFlk-1, mFlt-1, mTie2, mVE-cadherin, mCD31, mCD34, mCD45, mTER (an erythroid marker) and mMac-1 (CD11b; myeloid marker). Resected xenografts were passed three times through a 200 μm gauge^−1^ stainless steel mesh after being minced. The cells were suspended in a medium containing a 20–60% Percoll™ gradient (Amersham Pharmacia Biotech, Uppsala, Sweden) and centrifuged at 1500 r.p.m. for 20 min at room temperature. The cells in the 30% layer of Percoll were then collected, and erythrocytes were removed by treatment with 0.83% ammonium chloride in 10 mM Tris-HCl (pH 7.5). Peripheral blood was centrifuged on lymphosepar 2 (Immuno-Biological Laboratories, Gunma, Japan) at 1800 r.p.m. for 30 min at room temparature after red cell lysis and the cells were collected as MNCs. Bone marrow from the femur was also treated with 0.83% ammonium chloride in 10 mM Tris-HCl (pH 7.5) for red cell lysis and subjected to flow cytometric analysis. Rat anti-murine (m) CD31, biotin-conjugated rat anti-mCD34, rat anti-mCD45, rabbit anti-mFlt-1, phycoerythrin(PE)-conjugated rat anti-mFlk-1, rabbit anti-mTie-2, goat anti-mVE-cadherin, rat anti-mTER119, and rat anti-mMac-1 (BD Pharmingen, San Diego, CA, USA) were used as the primary antibodies. Anti-rat fluorescein isothiocyanate (FITC), anti-rabbit FITC, anti-goat FITC and anti-rat-phycoerythrin (PE), streptavidin-peridinin chlorophyll-a Protein(PerCP) (BD Pharmingen, San Diego, CA, USA) were used as the secondary antibodies. All primary antibodies were subjected to isotype control. To analyse the EPC or EC population, we gated on the lymphocyte-size fraction (Murohara *et al*, 2000). EPCs(mCD34+, mFlk-1+) in the BM,PB and TI were enumerated by three-colour flow cytometry to detect the expression of mFlt-1, mTie-2, mVE-cadherin, mCD31.

### EPC culture

On day 1 of culture, BM- or PB-derived EPCs were cultured in Stem pro (GIBCO BRL, Grand Island, NY, USA). Floating MNCs and sorted mCD34-positive MNCs were cultured overnight on non-coated plastic plates, and 5×10^5^ cells were then transferred to murine fibronectin-coated plastic plates (GIBCO BRL) for culturing. From day 2 of culture, EPCs were cultured in SFM (GIBCO BRL) supplemented with 20% FBS and bovine pituitary extract. On days 4 and 13 of culture, AT cells and clusters were examined. On day 13, numbers of AT cells in each BM and PB sample were counted.

### Immunocytochemistry

Spindle-shaped AT cells observed at 14 days of culture were subjected to immunocytochemistry to analyse the expression of Mac-1-FITC (Pharmingen).

### Cellular uptake of acetylated LDL

We investigated Ac-LDL uptake (a process characteristic of endothelial lineage) into AT cells (Asahara *et al*, 1997). AT cells cultured on fibronectin were incubated in medium containing 10 μg ml^−1^ DiI-labelled Ac-LDL (DiI-Ac-LDL; Molecular Probes, Eugene, OR, USA) for 24 h at 37°C. Cells were then examined under a fluorescence microscope.

### Culture of EC and EPC on HUVEC monolayer

BM-derived MNCs from WIBC-9 xenografted mice that are mCD34^+^ and mFlt-1^+^ (sorted by FACS), were cultured in Stem pro (GIBCO BRL, Grand Island, NY, USA) for 13 days and incubated in the medium containing 10 μg ml^−1^ DiI-labelled Ac-LDL (DiI-Ac-LDL; Molecular Probes, Eugene, OR, USA) for 24 h at 37°C. These cells were seeded on HUVEC monolayer cultured on Growth Factor Reduced Matrigel matrix (Becton Dickinson Labware, Bedford, MA) on day 5.

### Preparation of BM derived CD34^+^+Flk-1^+^ subset and CD34^+^+Flk-1^−^ subset cultured conditioned medium and determination of mVEGF and mIL-8 concentration by ELISA

BM derived CD34^+^+Flk-1^+^ MNC subset and CD34^+^+Flk-1^−^ MNC subset (1×10^5^ cells well^−1^) were sorted by using FACS (*n*=5) and incubated for 14 days at 37°C, and the supernatants were collected every 12 h and stored at −80°C until used. Concentrations of mVEGF and mIL-8 were measured by ELISA in 100 μl samples of supernatant, using Immunoassay Kits (Immuno-Biological Laboratories Co. Ltd., Fujioka, Japan). Each assay was performed in triplicate.

### Statistical analysis

All data are expressed as the mean±s.d. StatView computer software (ATMS Co., Tokyo, Japan) was used for the statistical analysis of differences in results of MTT, migration assay and EPC population between groups. Two-sided *P*<0.05 and *P*<0.01 were considered to indicate statistical significance.

## RESULTS

### Flow cytometry (quantitation of EPCs) (Table 1 and Figures 1 and 2)

**Table 1:**
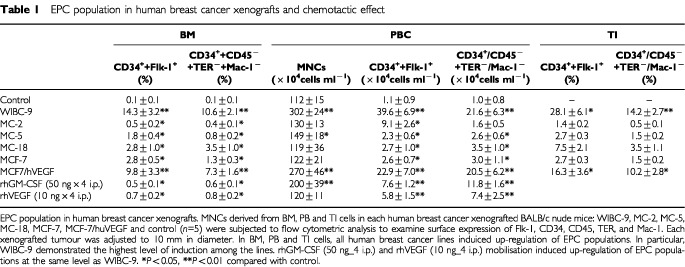
EPC population in human breast cancer xenografts and chemotactic effect

**Figure 1:**
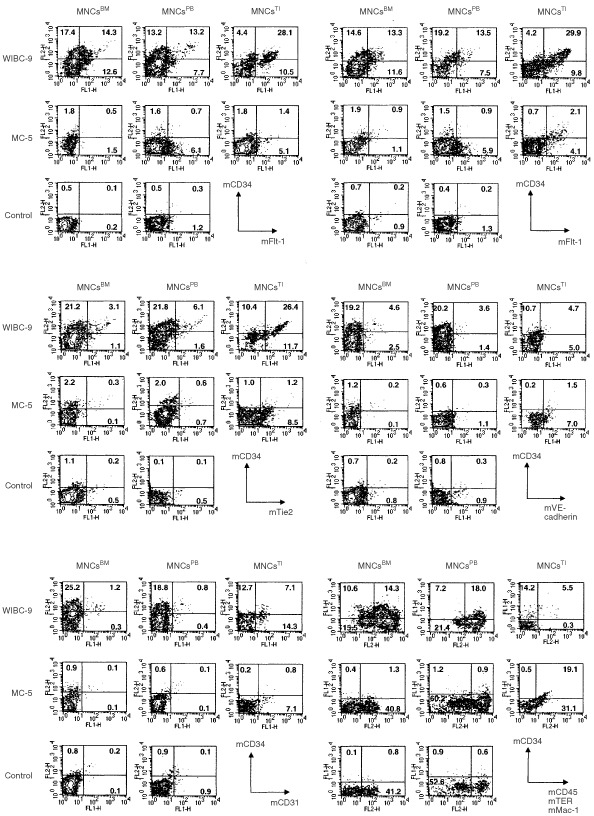
Flow cytometry (quantitation of EPCs and ECs). In WIBC-9 xenografted mice, the populations of EPCs were markedly elevated in MNCs derived from BM, PB and TI cells. The following MNC subtypes were clearly visualised: CD34^+^/Flk-1^+^, CD34^+^/Flt-1^+^, CD34^+^/Tie2^+^ and CD34^+^/VE-cadherin^+^. In WIBC-9 mice, the population of cells that were CD45^−^/TER^−^/Mac-1^−^/CD34^+^ was approximately 10%, almost equal to the population of cells that were CD34^+^/Flk-1^+^ MNCs. The EC population (CD34^+^/CD-31^+^ MNCs) was also high in WIBC-9 mice, although not as high as the EPC population. EC populations of MC-5 xenografted mice were higher than those of the controls.

**Figure 2:**
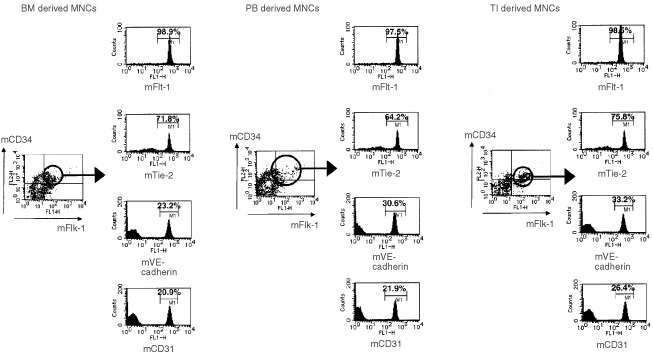
EPCs(mCD34+, mFlk-1+) in the bone marrow and peripheral blood in WIBC-9, included a large number of FLT1+(98.9%/97.5%), and TIE2+(71.8%/64.2%) but include small number of VE-cadherin (23.2%/30.6%) and CD31+(20.9%/20.9%).

The population of EPCs was markedly elevated in all three sources of MNCs (BM, PB and TI) in WIBC-9 xenografted mice. The MNC subtypes that were clearly visualised were CD34-positive (CD34^+^)/Flk-1^+^, CD34^+^/Flt-1^+^, CD34^+^/Tie2^+^ and CD34^+^/VE-cadherin^+^. It should be noted that, in WIBC-9 mice, the population of cells that were CD45-negative (CD45^−^)/TER119^−^/Mac-1^−^/CD34^+^ (considered part of the EPC population) was around 10%, which is almost equal to the number of CD34^+^/Flk-1^+^ MNCs in BM and PB. The EC population (CD34^+^/CD31^+^ MNCs) was also prominent in WIBC-9 mice. The real number of MNCs in PB was also elevated in WIBC-9, MCF-7/hVEGF, and rhGM–CSF mice. EPC and EC populations were slightly elevated in MC-5. These results are shown in Table 1, with additional data for MC-2, MC-18, MCF-7 and MCF-7/hVEGF, plus data for chemotactic effects. All human breast cancer lines induced EPC populations in BM, PB compared with control. WIBC-9 induced the highest EPC populations (10% or greater) among the breast cancer lines. rhGM–CSF (50 ng×4, i.p.) and rhVEGF (10 ng×4, i.p.) also induced significantly higher levels of EPC populations only in peripheral blood compared with control. EPCs(mCD34+, mFlk-1+) in the BM,PB and TI enumerated by three-color flow cytometry showed high expression of mFlt-1 and low expression of mCD31.

### AT cell population in samples from xenografted mice (Figure 3A)

**Figure 3:**
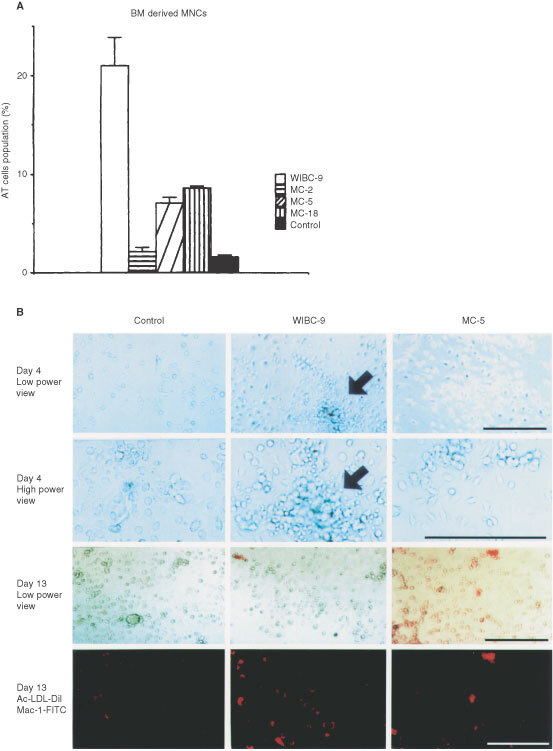
(**A**) AT cell population in the xenografts. AT cells, including ECs were counted. In WIBC-9 samples, the AT cell population was high. AT populations in other cancer lines were higher than in the control, a finding consistent with the size of the CD34^+^/Flk-1^+^ MNC population. (**B**) BM-derived EPC morphogenesis on murine fibronectin. On day 4 after plating CD34^+^ floating MNCs, spindle-shaped AT cells and cluster formation were evident in WIBC-9 samples. On day 13, all AT cells were found to uptake DiI-labelled acLDL. Immunocytochemistry revealed that all cells were Mac-1–negative. Each scale bar is 50 μm.

In WIBC-9 samples, AT cells comprised markedly high percentages of total cells (20% or greater). In other lines, AT cell populations were higher than that of the control, a finding consistent with the observed CD34^+^/Flk-1^+^ populations.

### BM-derived EPC morphogenesis on murine fibronectin (Figure 3B)

On day 4 after plating the CD34^+^ floating MNCs, spindle-shaped AT cells and cluster formation were clearly visible in WIBC-9 samples. On day 13, WIBC-9 AT cells were found to uptake DiI-labelled acLDL. Immunocytochemistry revealed that these cells were negative for Mac-1, indicating that they were not of a monocyte lineage.

### Culture of EC and EPC on HUVEC monolayer (Figure 4)

**Figure 4:**
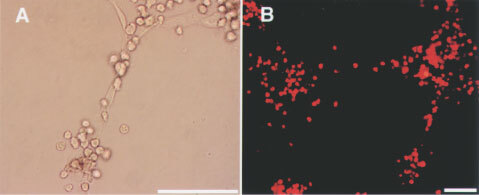
(**A**) HUVECs and BM-derived EPCs (mCD34^+^ and mFlt-1^+^) formed capillary networks. (**B**) BM-derived EPCs, which incorporated DiI-labelled acLDL, were integrated with HUVECs. Phase contrast photomicrographs showed capillary networks. Each scale bar is 10 μm.

BM-derived MNCs that are mCD34^+^ and mFlt-1^+^ incorporated DiI-labelled acLDL and were integrated in a HUVEC monolayer (forming capillary networks) implying their maturation to ECs.

### The production of angiogenic factors by putative EPCs (Figure 5)

**Figure 5:**
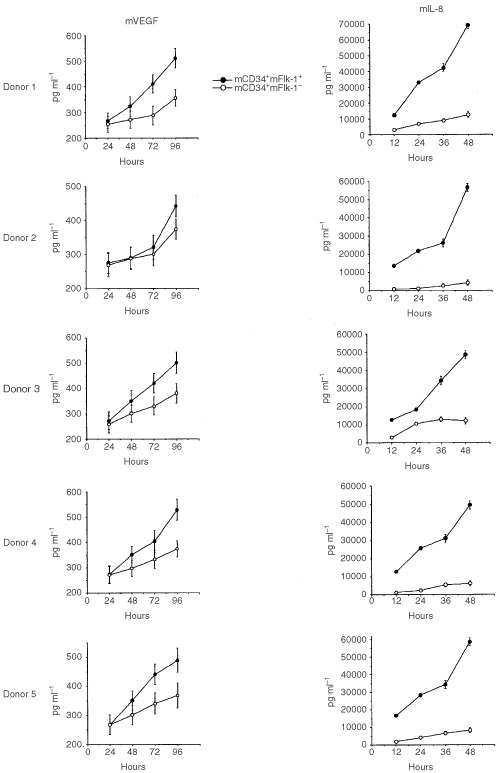
BM derived CD34^+^+Flk-1^+^ MNC subset and CD34^+^+Flk-1^−^ MNC subset (1×10^5^ cells well^−1^) were sorted by using FACS (*n*=5) and incubated for 14 days at 37°C, and the supernatants were collected every 12 h and stored at −80°C until used. Concentrations of mVEGF and mIL-8 were measured by ELISA in 100 μl samples of supernatant, using Immunoassay Kits (Immuno-Biological Laboratories Co., Ltd., Fujioka, Japan). Each assay was performed in triplicate.

BM derived CD34^+^Flk-1^+^ MNC subset and CD34^+^Flk-1^−^ MNC subset produced mVEGF and mIL-8 on time course. The former subset demonstrated significantly higher levels of murine VEGF and IL-8 in culture supernatant on time course by ELISA.

## DISCUSSION

Neovascularisation encompasses both angiogenesis and vasculogenesis. In angiogenesis (the classic paradigm for new vessel growth), mature, differentiated ECs break free from their basement membrane, migrate and proliferate to form branches of parental vessels. The fact that tumours induce angiogenesis indicates that tumour development and metastasis are dependent upon neovascularisation, and suggests that this relationship involves angiogenic growth factors that are specific to neoplasms (Folkman and Klagsbrun, 1987; Kendall and Thomas, 1993; O'Reilly *et al*, 1997; Goldman *et al*, 1998; Tanaka *et al*, 1998; Ferrara and Alitalo, 1999; Mori *et al*, 2000). Although the therapeutic potential of anti-angiogenic factors is reportedly promising, their antiangiogenic mechanisms (inhibition of migration, proliferation and tube formation) have not been well characterised. Vasculogenesis involves participation of BM-derived EPCs that circulate to sites of neovascularisation, where they differentiate into mature ECs (Asahara *et al*, 1997, 1999; Isner and Asahara, 1999; Takahashi *et al*, 1999; Kalka *et al*, 2000; Murohara *et al*, 2000). The results of the present study indicate that six human breast cancer lines, including the IBC line WIBC-9, induce postnatal EPC kinetics as well as EC kinetics. The chemotactic modulation of proliferation and migration of EPCs that we observed was mainly the result of VEGF mobilisation, a finding consistent with those of a previous study (Asahara *et al*, 1997). MCF-7/hVEGF and WIBC-9 showed markedly enhanced growth on mice and higher microvascular density by histology compared with MCF-7 in this series study (Shirakawa *et al*, 2001) as previously described (Lewin *et al*, 1999). This enhanced growth may be a result of contribution of endothelial precursors induced by angiogenic factor such as VEGF (de Bont *et al*, 2001). Moreover, MNCs which contain haematopoietic precursors as well as EPCs in peripheral blood markedly increased in WIBC-9 and MCF7/hVEGF xenografted mice. This may be due to the mobilisation of the common precursors for EPCs and haematopoietic cells residing in bone marrow led by the elevation of plasma levels of factors such as VEGF secreted by cancer cells (Hattori *et al*, 2001). Thus, our results support the notion that BM-derived precursors provide a sufficient source of ECs to enhance the growth of breast cancer *in vivo*. *In vitro*, the CD34^+^ floating MNCs on day 4 after plating, spindle-shaped AT cells and cluster formation were clearly visible in WIBC-9 samples. Although, we have already reported the TI EPCs in WIBC-9 xenografted mice(Shirakawa *et al*, 2002), the population of EPCs was markedly elevated in all three sources of MNCs (BM, PB and TI) in WIBC-9 among the xenografted mice. These facts possibly show the induction of vasculogenesis especially in WIBC-9. On day 13, AT cells from WIBC-9 were found to uptake DiI-labelled acLDL. Immunocytochemistry revealed that these cells were negative for Mac-1, indicating that they were not of a monocyte lineage. These facts possibly show the maturation from EPC to EC *in vitro* culture.

In WIBC-9, human angiogenic factors (*hAng1*, hVEGF, hbFGF) and murine angiogenic factors (mflt-1, m integrin β3, mVEGF, and mCD31), were expressed at higher levels than they were in the three non-IBC xenografts. However, because our results indicate that the human breast cancer lines MC-2, MC-5 and MC-18 secrete low levels of hVEGF, modulation by accessory molecules constitutes a cytokine network, and autocrine or paracrine secretion of angiogenic factors is likely to be associated with modulation of EPC recruitment (Shirakawa *et al*, 2001). The *VEGF* family receptor, *mflt-1* and the Ang receptor, *hTie-1*, and *hTie-2*, *mtie-2* was expressed at a higher level in WIBC-9, but *mflk-1* and *mflt-4* were not detected in WIBC-9. Expression of *hFlt-1* and *KDR* was detected in all xenografts. The cytokine, *hIL-1*β was only detected in WIBC-9, and a higher expression of *hIL-8* was detected in WIBC-9. The adhesion molecule, *h* *integrin* β*3*, *m* *integrin* β*3* and *m* *integrin* α*v* were detected at higher levels in WIBC-9 as previously described (Shirakawa *et al*, 2001).

BM-, PB- and TI-derived murine EPCs and ECs from human breast cancer-bearing mice were found to exhibit a specific pattern of cell surface antigen expression; i.e., double positive for mCD34 and mFlk-1. The subtypes, which may show the maturation of EPC, detected were mCD34^+^/mFlk-1^+^, mCD34^+^/mFlt-1^+^, mCD34^+^/mTie-2^+^, mCD34^+^/mVE-cadherin^+^ and mCD34^+^/mCD31^+^. In this model, we detected both EPCs and ECs, or their subtypes (maturation), among BM-, PB- and TI-derived cells. The data of multiple markers analysis, which are Flk-1, Flt-1, Tie-2, VE-cadherin, and CD31 by using tri-color FACS, indicated that the populations of CD34^+^+FLK1^+^ in the bone marrow and peripheral blood in WIBC-9, included a large number of FLT1+(98.9%/97.5%), and TIE2+(71.8%/64.2%) but include small number of VE-cadherin (23.2%/30.6%) and CD31+(20.9%/20.9%). Induced EPCs and ECs were clearly shown to be non-malignant cells, and May-Gimsa staining revealed that they were of the same phenotype as rhVEGF- and rhGM–CSF–mobilised EPCs and ECs (data not shown). Surprisingly, induced EPC populations which were positive of mCD34 and mFlk-1, showed the production of mVEGF and mIL-8 in culture supernatant on time course. This result implies autocrine regulation of proliferation of these precursors *in vivo*, and coincides with the recent report of the regulation of haematopoietic stem cells (Gerber *et al*, 2002). In our model, this autocrine cascade may play an important role of the induction of vasculogenesis and the tumour growth.

The chemotactic expansion of EPCs derived from PB or cord blood and transplantation of these EPCs have previously been reported (Asahara *et al*, 1997, 1999; Isner and Asahara, 1999; Takahashi *et al*, 1999; Kalka *et al*, 2000; Murohara *et al*, 2000); transplanted EPCs were found to significantly proliferate in ischaemic foci and differentiate *in situ* through a process of vasculogenesis. In the present study, we demonstrated that breast cancer-bearing mice exhibit significant expansion of precursors including EPCs and ECs; specifically, maturation and proliferation of these cells in tumours was clearly evident.
